# Elderly patients in psychiatry: Evolution and social challenges after a first hospitalization at an advanced age

**DOI:** 10.1192/j.eurpsy.2025.882

**Published:** 2025-08-26

**Authors:** D. Mezri, A. Gharbia, S. Hamzaoui, R. Hosni, U. Ouali, A. Aissa, R. Jomli

**Affiliations:** 1 Avicenne, Razi, Mannouba, Tunisia

## Abstract

**Introduction:**

Due to longer life expectancies, improvements in psychiatry, and reduced stigma about mental health in Tunisia, we are seeing more elderly people hospitalized for the first time in our department. Caring for this group presents both social and clinical challenges. While we are getting better at handling the clinical aspects, we often forget the social factors, which are crucial for the progress, follow-up, and mental well-being of older patients. These social issues can also lead to relapses or new mental health problems in this population.

**Objectives:**

Determine the evolution of elderly patients hospitalized for the first time in the psychiatric department.

**Methods:**

This is a retrospective study. We examined the records of all patients aged over 65 who were admitted to the Avicenne Psychiatric Department at Razi Hospital from September 2022 to September 2024.

**Results:**

A total of 22 patients were identified, including 16 men and 6 women. The average age of these patients was 68 years, with ages ranging from 64 to 80 years. The majority came from an urban background (81%). The educational level of our patients was primary at 19%, an university level at 34% and a secondary school education at 47%. Among our patients, 13% are still professionally active and 57% retired. The socioeconomic level of our patients was affluent in 42%, average in 34%, and low in 24%. Within our patient population, 61% were married, 24% divorced, 10% were single and 4% widow or widower. 23% of our patients were living alone and 77% were living with one or more family members. Concerning the diagnosis, we observed mental confusion caused by an organic pathology in 9%, a purely neurological cause of the disorder in 14% ( dementia in 10% and Parkinson’s disease in 4%), a depressive episode in 31%, a manic episode within the context of bipolar disorder in 37%, and schizophrenia in 9%. During hospitalization, 27% of the patients experienced family rejection from their relatives. After leaving the hospital, a significant number of patients were lost to follow-up (63%), 22% had regular follow-up and 13% attended appointments irregularly.

**Conclusions:**

This study shows that more elderly patients are being hospitalized for the first time in psychiatry. Many face social challenges, such as family rejection and losing contact after leaving the hospital.The creation of retirement homes and social support systems may be a solution to provide safe living and support for these patients, helping them stay healthy and connected after hospitalization.

**Disclosure of Interest:**

None Declared

